# Lessons from a Single Amino Acid Substitution: Anticancer and Antibacterial Properties of Two Phospholipase A_2_-Derived Peptides

**DOI:** 10.3390/cimb44010004

**Published:** 2021-12-22

**Authors:** José R. Almeida, Bruno Mendes, Marcelo Lancellotti, Gilberto C. Franchi, Óscar Passos, Maria J. Ramos, Pedro A. Fernandes, Cláudia Alves, Nuno Vale, Paula Gomes, Saulo L. da Silva

**Affiliations:** 1 Universidad Regional Amazónica Ikiam, Km 7 Via Muyuna, Tena 150150, Ecuador; rafael.dealmeida@ikiam.edu.ec (J.R.A.); bruno000mendes@gmail.com (B.M.); biomol2@hotmail.com (S.L.d.S.); 2 Departmento de Bioquímica e Biologia Tecidual, Instituto de Biologia, Universidade Estadual de Campinas (UNICAMP), Campinas 13083-862, SP, Brazil; marcelo.lancellotti@fcf.unicamp.br; 3 Faculdade de Ciências Farmacêuticas, Universidade Estadual de Campinas (UNICAMP), Campinas 13083-871, SP, Brazil; 4 Centro Integrado de Pesquisas Oncohematologicas da Infancia, Universidade Estadual de Campinas (UNICAMP), Campinas 13083-881, SP, Brazil; gfran@unicamp.br; 5 LAQV/REQUIMTE, Departamento de Química e Bioquímica, Faculdade de Ciências, Universidade do Porto, Rua Campo Alegre s/n, 4169-007 Porto, Portugal; oscar.passos@fc.up.pt (Ó.P.); mjramos@fc.up.pt (M.J.R.); pafernan@fc.up.pt (P.A.F.); claudialves05@gmail.com (C.A.); pgomes@fc.up.pt (P.G.); 6 OncoPharma Research Group, Center for Health Technology and Services Research (CINTESIS), Rua Doutor Plácido da Costa, 4200-450 Porto, Portugal; 7 Department of Community Medicine, Information and Health Decision Sciences (MEDCIDS), Faculty of Medicine, University of Porto, Alameda Professor Hernâni Monteiro, 4200-319 Porto, Portugal; 8 Faculty of Chemical Sciences, University of Cuenca, Cuenca 010107, Ecuador

**Keywords:** *Agkistrodon*, leucine, membrane, phenylalanine, venom peptides

## Abstract

The membrane-active nature of phospholipase A_2_-derived peptides makes them potential candidates for antineoplastic and antibacterial therapies. Two short 13-mer C-terminal fragments taken from snake venom Lys49-PLA_2_ toxins (p-AppK and p-Acl), differing by a leucine/phenylalanine substitution, were synthesized and their bioactivity was evaluated. Their capacity to interfere with the survival of Gram-positive and Gram-negative bacteria as well as with solid and liquid tumors was assessed in vitro. Toxicity to red blood cells was investigated via in silico and in vitro techniques. The mode of action was mainly studied by molecular dynamics simulations and membrane permeabilization assays. Briefly, both peptides have dual activity, i.e., they act against both bacteria, including multidrug-resistant strains and tumor cells. All tested bacteria were susceptible to both peptides, *Pseudomonas aeruginosa* being the most affected. RAMOS, K562, NB4, and CEM cells were the main leukemic targets of the peptides. In general, p-Acl showed more significant activity, suggesting that phenylalanine confers advantages to the antibacterial and antitumor mechanism, particularly for osteosarcoma lines (HOS and MG63). Peptide-based treatment increased the uptake of a DNA-intercalating dye by bacteria, suggesting membrane damage. Indeed, p-AppK and p-Acl did not disrupt erythrocyte membranes, in agreement with in silico predictions. The latter revealed that the peptides deform the membrane and increase its permeability by facilitating solvent penetration. This phenomenon is expected to catalyze the permeation of solutes that otherwise could not cross the hydrophobic membrane core. In conclusion, the present study highlights the role of a single amino acid substitution present in natural sequences towards the development of dual-action agents. In other words, dissecting and fine-tuning biomembrane remodeling proteins, such as snake venom phospholipase A_2_ isoforms, is again demonstrated as a valuable source of therapeutic peptides.

## 1. Introduction

Bioactive peptides have opened a new horizon in drug discovery and are currently considered a cornerstone in developing therapies for cancer and bacterial infections [1]. In the last years, more than 7% of Food and Drug Administration-approved drugs are peptide-based entities [2]. Yet, the design and refinement of these active structures are highly challenging [3], and several prediction models and tools are being proposed and have been made available in recent years [4,5]. However, despite these bioinformatics and statistical advances, the isolation of peptides from natural sources, the identification by genomic and transcriptomic investigations, or the synthesis of molecular region mimics of target proteins remain useful both to discover new structures and to understand functional aspects that are crucial for the selection of more potent and selective molecules [6,7].

Toxins are natural products characterized by experiencing critical selective pressures for their action in pathways and cell structures of biomedical interest [8]. In snake venoms, a diversity of protein isoforms is found [9]. This repertoire of molecules with subtle differences in the primary structure offers ample opportunity to find therapeutic applications and obtain relevant information to elaborate predictive models and synthesize more efficient molecules. Biomedical products derived from toxins are a classical example of the pivotal contribution of natural products in the drug discovery process. An important precedent is Captopril, an antihypertensive synthetic analogue of one short molecule from *Bothrops jaracaca*, which is considered the first member of angiotensin-converting enzyme inhibitor medications [10]. In this connection, snake venom phospholipases A_2_ (PLA_2_) are a diverse group of great pharmaceutical relevance [11]. The PLA_2_ family is extremely capable of interacting, modifying, and disrupting membrane lipids [12,13]. Additionally, the membranes of pathogens and cancer cells are an attractive target for low-resistance therapeutic approaches [14]. Thus, many lytic PLA_2_-derived peptides with antitumor [15,16], leishmanicidal [17], and antibacterial [18,19] effects have been synthesized to reproduce biological interactions between the C-terminal of the protein templates and biomembranes. The peptide p-AppK (KKYKAYFKLKCKK) is a specific example of that, inspired in the myotoxic C-terminal region of a Lys49-PLA_2_ derived from the snake *Agkistrodon piscivorus piscivorus*. This short synthetic molecule has antitumoral activity against murine tumor cell lines, such as sarcoma, melanoma, mammary carcinoma, and others [15]. p-AppK is amongst the PLA_2_-mimicking peptides with highest cytotoxic activity, higher than that of pEM-2 that is recognized by its in vivo action [15]. Curiously, the 115–129 C-terminal region from the snake *Agkistrodon contortrix laticinctus*, corresponding to peptide p-Acl (KKYKAYFKFKCKK), has high sequence identity with p-AppK, differing only in the amino acid residue at position 9 (where p-Acl has a phenylalanine in place of the leucine present in p-AppK). Peptide p-Acl has not been previously evaluated against either bacteria or cancer cells, whereas some authors have reported that the presence of phenylalanine in certain peptide sequences is a determinant factor for the display of anticancer and antibacterial activities. Phenylalanine-containing peptides selectively recognize and fuse to certain membranes [20,21]. In view of this, we synthesized p-AppK and p-Acl for comparative evaluation of their cytotoxic and antibacterial activities, in order to establish structure–activity relationships (SAR) that can guide the bioengineering of novel peptides with dual therapeutic activity. These were further simulated in silico, to obtain a molecular-level picture of the effects induced by the peptides upon interaction with bacterial and eukaryotic (healthy and cancerous) cell membranes. Results herein reported confirm both peptides as dual-action therapeutic leads, where the Leu→Phe substitution, from p-AppK to p-Acl, apparently leads to an increase in peptide’s antibacterial and antiproliferative potency.

## 2. Materials and Methods

### 2.1. Sequence Analysis

Online bioinformatics tools were used to perform amino acid sequence analysis of the peptides. A functional study was first run using CAMPR3 (http://www.camp.bicnirrh.res.in/prediction.php, accessed on 1 December 2021). AntiCP (https://webs.iiitd.edu.in/raghava/anticp/index.html, accessed on 1 December 2021), ACPred (http://codes.bio/acpred/, accessed on 1 December 2021), and CPPred-RF (http://server.malab.cn/CPPred-RF/index.jsp, accessed on 1 December 2021) were also used to predict the antimicrobial, anticancer, and cell-penetrating properties of both peptides, respectively. The Rational Design of Antimicrobial Peptides tool in CAMPR3 (http://www.camp.bicnirrh.res.in/predict_c21/, accessed on 1 December 2021) was next employed to generate peptide analogues to confirm whether the exchange of amino acids found in nature is predicted as favorable to the antimicrobial activity by different software algorithms (Support Vector Machine, Random Forest, and Discriminant Analysis classifiers). Finally, PepDraw (http://www.tulane.edu/~biochem/WW/PepDraw/, accessed on 1 December 2021) was used to calculate the peptides’ total net charge, PI and hydrophobicity, taking into account the C-terminal amidations.

### 2.2. Peptide Synthesis

C-terminally amidated p-AppK and p-Acl peptides were assembled in a CEM Liberty 1 system employing a rapid MW-SPPS protocol on Rink amide-MBHA resin. Once the elongation of the polypeptide chain ended, the peptide was fully deprotected and cleaved from the resin by a 2 h acidolytic treatment, at room temperature (RT), using a cocktail containing 95% trifluoroacetic acid (TFA), 2.5% triisopropylsilane (TIS), and 2.5% water. The crude peptides thus obtained were precipitated with cold diethyl ether, centrifuged, and the peptide pellets collected, re-dissolved in 10% aqueous acetic acid, and lyophilized. The peptides were then purified by RP-MPLC using the same conditions described by Almeida et al. (2018) [19]. The purity degree of peptides was determined by reverse-phase high performance liquid chromatography (RP-HPLC), and the purification step repeated, if necessary, until a minimum purity of 95% was reached. Finally, the molecular identity of the pure peptides was confirmed by ESI-IT MS, using an LTQ OrbitrapTM XL hybrid mass spectrometer.

### 2.3. Hemolysis Assays

The erythrocyte-lysing potential of the peptides was analyzed by in silico and in vitro approaches. The hemolytic peptide modules of the following bioinformatics tools: HLPred-Fuse (http://thegleelab.org/HLPpred-Fuse/, accessed on 1 December 2021), HAPPENN (https://research.timmons.eu/happen, accessed on 1 December 2021), HEmoPI (https://webs.iiitd.edu.in/raghava/hemopi/, accessed on 1 December 2021), and HempPImod (https://webs.iiitd.edu.in/raghava/hemopimod/ter_str.php, accessed on 1 December 2021) were used to predict the effect on the RBC. Quantitative in vitro hemolytic activity assays against heparinized collected human RBC (type AB Rh-) were performed as previously reported by Proaño-Bolanos et al., 2019 [22]. Briefly, a suspension of the mammalian cells was incubated with different concentrations of peptides dissolved in PBS buffer for 2 h at 37 °C. After this procedure, the material was centrifuged, and the supernatant aliquoted to a 96-well microplate. The percentage of disrupted RBC was quantitated by spectrophotometry, based on absorbance readings at 405 nm using a VersaMax multiwell plate reader (Molecular Devices, Sunnyvale, CA, USA). Erythrocytes incubated with Triton 2%, a known membrane-damage agent, were taken as positive control (100% hemolysis). Hemolytic assays were performed in triplicates at least three times.

### 2.4. Antibacterial Activity

The broth microdilution method was used to determine whether p-AppK and p-Acl display antibacterial properties. This evaluation was performed on three Gram-negative (*Pseudomonas aeruginosa* 31NM, *P. aeruginosa* ATCC 27853, and *Escherichia coli* ATCC 25922) and two Gram-positive (*Staphyloccocus aureus* BEC9393 and *S. aureus* Rib1) bacterial strains. The bacterial growth inhibition protocols were as detailed in a previous report from our group [19]. Briefly, the mid-log phase bacterial growth suspensions were incubated for 24 h with the synthetic peptides in a 96-well microplate. After this period, bacterial growth was determined, considering the absorbance values at 595 nm. Optical density measurements of bacterial suspensions incubated in medium only were taken as 100% growth. Three independent experiments were carried out in triplicate.

### 2.5. Membrane Damage

The effect of p-AppK and p-Acl on the membranes of Gram-positive (*S. aureus* BEC9393) and Gram-negative (*P. aeruginosa* ATCC 27853) bacteria was investigated using propidium iodide (PI) uptake assays. The bacteria were cultured and exposed to 100 µM peptides and PI (15 µg/mL). Aliquots (200 µL) of the test solutions were added to 96-well microplates. The time-dependent changes in PI fluorescence were quantified and recorded at excitation and emission wavelengths of 580 and 620 nm, respectively, for 30 min, using a VersaMax fluorescence microplate reader (Molecular Devices, Sunnyvale, CA, USA). The membrane damage was assessed by three independent assays performed in triplicate.

### 2.6. Cytotoxicity

In vitro cell viability assays were conducted to screen the anticancer properties of peptides p-AppK and p-Acl. The assays were based on colorimetric detection of the enzymatic reduction of 3-[4,5-dimethylthiazole-2-yl]-2,5-diphenyltetrazolium bromide (MTT) that occurs in viable cells. A vast repertoire of 13 solid tumors and 10 leukemia cell lines from the cell bank of the Integrated Center for Childhood Onco-Hematological Investigation (UNICAMP, Brazil) was used. Namely, the following solid malignant neoplasms were used: OVCAR (human ovarian adenocarcinoma), MACL1 and MGSO3 (human primary breast cancer), MCF7 (human breast adenocarcinoma), VW473 (human medulloblastoma), MG63 (human osteosarcoma), SHSY5S (human neuroblastoma), NCI (non-small cell lung cancer), U138 (human neuronal glioblastoma), U87 (glioblastoma), PC3 (human prostate cancer), H1299 (human non-small cell lung carcinoma), and HOS (human osteosarcoma). Leukemia cell lines included K562 (chronic myeloid/chronic myelogenous human), NB4 (human acute promyelocytic leukemia), HL60 (acute promyelocytic leukemia), RAMOS (human Burkitt lymphoma), NALM6 (acute B lymphoblastoma), B15 (human B cell precursor leukemia), REH (acute lymphocytic leukemia non-T; non-B), JURKAT (human T cell lymphoblast-like), TALL (acute lymphoblastic T-cell leukemia), and CEM (human acute lymphoblast leukemia). All procedures were run in automated mode on a Liquid Handling Workstation epMotion 5070 (Eppendorf). Firstly, the cell lines used in this investigation were cultured in plastic bottles (75 mL) in the presence of RPMI 1640 (Sigma R6504) medium supplemented with 10% fetal calf serum, 1% penicillin (IU/mL), and streptomycin (10 mg/mL) at 37 °C under humidified air with 5% CO_2_. The tumor cells were distributed in 96-well microplates and were exposed to 100 µM solutions of the synthetic peptides. The metabolic activity of cells was measured by spectrophotometric monitoring of the absorbance at 570 nm on a Synergy ELISA plate reader (Bio Tek Instruments). Two reference antineoplastic drugs, paclitaxel and vincristine, were used as positive controls, whereas cells growing in medium alone was taken as 100% viability (negative control). The results were expressed as cell viability inhibition and represent three independent experiments run in triplicate.

### 2.7. Molecular Dynamics Simulations

#### 2.7.1. Membrane Model

We modelled six different membranes of pure and mixed composition, each with either p-Acl or p-AppK peptides inserted across them, generating a total of twelve different systems. The membranes were inserted in rectangular boxes of water extending by 32 Å beyond the membrane headgroups. Counter-ions (K^+^ and Cl^−^) were used to neutralize the system (Supplementary Materials). 

The number of phospholipids per membrane was 140; the total number of atoms was 37,447–48,190, depending on the specific membrane simulated. The six membrane models were composed of: 100% 2,3-dioleoyl-d-glycero-1-phosphatidylserine (DOPS, membrane 1, a totally negative membrane, which might exist in small, specific regions of the eukaryotic membrane, and for which the peptides should have maximal affinity); 100% 2,3 dipalmitoyl-d-glycero-1-phosphatidylcholine (DPPC, membrane 2, representing the dominant zwitterionic composition of eukaryotic membranes); 86% 1-palmytoil-2-cis-9,10-methylenehexadecanoyl-phosphorylethanolamine (PMPE); 4% palmitic acid (PAL); 4% palmitoleic acid (PALO); 4% oleic acid (OLE); 2% myristic acid (MYR, membrane 3, whose composition was taken from Almeida et al., 2018); 60% PMPE; 31% 1-palmitoyl-2-oleoyl-sn-glycero-3-[phospho-rac-(1-glycerol) (POPG); 6% cardiolipin with head group charge −2 (PVCL2); and 3% tetraoleoyl cardiolipin with head group charge −2 (TLCL2, membrane 4, whose composition was taken from Sahoo et al., 2017). Thymocytes-like membrane constituted by 13 different lipidic components (membrane 5, whose composition was taken from van Blitterswijk et al. [23] and is detailed in Supplementary Materials Table S1); and finally, a leukemia-like membrane constituted by 15 different lipidic components (membrane 6, composition taken from Van Blitterswijk et al. and detailed at Supplementary Materials Table S2). In summary, membranes 1 and 2 represent the extremes of the spectrum of the composition of eukaryotic membranes, which have a mix of anionic and zwitterionic phospholipids in different percentages at different regions; membranes 3 and 4 model the *E. coli* membrane; and membranes 5 and 6 are models for thymus and leukemia cancer cell membranes, respectively.

#### 2.7.2. Molecular Dynamics Simulations

A series of molecular dynamics simulations were carried out within the GROMACS 2018 software package [24]. We conducted a 5000-step minimization for each membrane–peptide system to remove eventual tensions or clashes in the system’s structure. Subsequently, the systems were gradually heated to 303.5 K in a 100 ps simulation using the Berendsen thermostat [25]. The density and interactions of the system were equilibrated in a 1 ns long simulation in the isothermal-isobaric ensemble (NPT) with decreasing restraints forces. At last, a production run of 200 ns was conducted in the NPT ensemble with Berendsen barostat [25]. The cut-off for the Lennard-Jones interactions was set to 12 Å. Coulomb interactions were treated using the Particle Mesh-Ewald (PME) summation method [26], with a cut-off of 12 Å for the real part of the sum. The time step of numerical integration was 2 fs (details in Supplementary Materials Table S3).

## 3. Discussion

Peptide drugs constitute an emerging therapy occupying a prominent market niche in the pharmaceutical industry trends [27,28]. The peptide therapeutics market is progressing with very favorable prospects, recognized potential, recent successful approvals by regulatory agencies and economic expansion [2]. The years of evolution of these compounds reveal clues for the selection of new hits, and simultaneously, new data to refine the statistical and computational models of prediction [29,30]. The bioengineering and optimization of antibacterial and antitumor peptides have been automated today [31,32]. The development of tools, methods, and computational algorithms has allowed significant advances in the peptide design and the understanding of critical interactions for the mechanism of action of these molecules [33,34]. However, in parallel, screening from natural resources, such as animal venoms and secretions, continues to be of great interest to the scientific and pharmaceutical community [35]. Motivated by this, in the present work, we combined computational and experimental approaches to evaluate the potential dual effects of structurally similar peptides derived from Lys49 toxins from snake venoms.

p-AppK was one of the first peptides mimicking PLA_2_s proteins reported to display antitumor properties. This peptide is active against B16 (melanoma), EMT-6 (mammary carcinoma), S-180 (sarcoma), and P3X (myeloma), with IC50 values between 56 µM and 156 µM, being more potent against melanoma [15]. The present work extends the understanding of the spectrum of bioactivity of this peptide by investigating its capacity to inhibit bacterial growth, not characterized previously. This enabled confirmation of the dual-action of p-AppK, which is proven efficient against Gram-positive and Gram-negative bacteria, including the multidrug-resistant (MDR) clinical isolates *P. aeruginosa* 31NM, *S. aureus* rib1 and *S. aureus* BEC93. The present study additionally covered the characterization of peptide p-Acl, which differs from p-AppK in a single amino acid residue (Leu→Phe). Both peptides recapitulate short fragments derived from the C-terminal end of snake venom PLA_2_ isoenzymes, and p-Ac1 was found to retain the antibacterial activity profile of p-AppK. Relevantly, the parent PLA_2_s from which both peptides derive are catalytically inactive isoforms. Yet, they retain the ability to disturb bacterial cell membranes by a mechanism independent of the enzymatic hydrolysis dictated primarily by the leading role of cationic and hydrophobic residues in the protein C-terminal region [36]. Hence, it is herein demonstrated that such ability is conserved by the synthetic peptides mimicking this region, as a rapid influx of PI into Gram-positive and Gram-negative cells was detected upon peptide treatment. The uptake of this cell-impermeable DNA dye reveals that the structural and dynamic properties of bacterial membranes were significantly compromised in the presence of synthetic peptides, which was further supported by molecular dynamics simulations. Thus, the alteration of the integrity of specific layers of membranes induced by the 13-mer peptides should have functional consequences that can lead to loss of intracellular content and affect the survival of bacteria. Won and Ianoul (2009) have demonstrated the ability of pEM-2, a modified peptide also derived from a PLA_2_, to interact with membrane phospholipids and induce cell lysis, which has been reported to be dependent on the lipid composition of the bacterial membrane and directly affected by hydrophobic interactions [36,37]. In general, the detergent and membrane disruptive action has been widely used as a strategy by many antimicrobial peptides and is seen as a promise to transform such peptides into therapeutics based on targeting bacterial membrane phospholipids [38,39].

Low toxicity is a keystone for the translational success of candidate peptides. Importantly, the biomimetic peptides herein studied did not promote lysis of the erythrocyte membrane in biologically active concentrations, suggesting selectivity and confirming the functional predictions determined by bioinformatics analyses. In earlier works addressing PLA_2_ peptide mimics, similar results were found, confirming this type of synthetic peptides as non-hemolytic [18,40]. Although red blood cells are considered a classical model for the initial assessment of peptide toxicity, further works should consider the use of other healthy cells. The tunability and simple peptide synthesis enable an easy fine-tuning, which contributes to selectivity and generation of safer and efficient analogues [28].

A selective molecular recognition ability was also suggested by the cytotoxicity assays. Despite promising activity in a wide variety of cancer cells, both peptides were inactive or showed low activity against some solid tumor cell lines, such as U138, MGSO3 and VW473. In agreement with this selective behavior, different profiles were obtained for leukemic cells as both peptides were strongly active against leukemic cells (particularly NB4, RAMOS and CEM). This selectivity is probably due to the diversity in the complex composition of membrane lipids, which are often suggested as primary targets for bioactive peptides [41], especially those derived from, or inspired in, membrane binding toxins. Cells that are similarly affected must have an analogous membrane lipid composition: for instance, p-Acl is more active in two lines of osteosarcoma cells (HOS and MG63), in line with the higher structural similarity between their respective membranes [42]. In view of this, the knowledge of the lipidome profile of different tumor cells might help to develop anticancer pharmaceuticals [43,44] acting via selective tumor cell membrane permeation. The molecular basis behind antitumor effects detailed here has not been experimentally accessed. However, it is likely based on lytic activity as evidenced for antibacterial action and reported for other anticancer peptides [45,46]. Additionally, our in silico approach suggested a possible cell-penetrating capacity, which may be in line with the antitumor activity and should be confirmed in the future. The molecular dynamics simulations shown here provide further support for this hypothesis, as the peptides induce rapid and spontaneous membrane permeability. These observations can lead to applying these peptides as tumor-cell-targeting carriers, facilitating the transport of impermeable drugs into the cell, hence enabling their action and clinical application. Combining these anticancer peptides with clinical drugs can also be an exciting route for the generation of combined therapies that potentiate antineoplastic effects and allow a fast and efficient clinical response. Oncolytic peptides have shown promising in vivo effects, being of relevance for the clinical development of a new generation of antineoplastic drugs [47]. Further studies should access the mode of action of p-AppK and p-Acl and evaluate the activity of scrambled versions to understand better the differences in cell recognition, especially in solid tumor cells.

Overall, this work confirms dual functionality for both p-AppK and p-Ac1, which highlights how membrane-binding toxins such as PLA_2_s are potential starting points to obtain short molecules capable of targeting cancer and bacterial cell membranes. Other previous studies have also reported potent antibacterial and antineoplastic peptides structurally related to p-AppK and p-Acl, such as pEM-2 [15], pep-MTII [16], pBmje [48] and pepBthTX-I [49]. Interestingly, these peptides were inspired in isoforms of membrane-remodeling proteins whose C-termini are highly homologous to those of our template proteins. As expected, p-AppK and p-Acl exhibit similar physicochemical properties, which also closely resemble those of other anticancer and/or antimicrobial peptides derived from PLA_2_s, namely the synthetic peptides mentioned above. Thus, all these peptides are highly cationic, possessing high pI values and several hydrophobic residues. Commonly, peptide charge and hydrophobicity have been highlighted as flagships in the development of antibacterial agents, taking into account the specific features of bacterial membranes [50]. Still, p-Acl generally demonstrated more significant activity against Gram-positive bacteria and some solid tumor cell lines, especially MG63 and HOS. Therefore, a single leucine→phenylalanine substitution translates into significant differences in the peptides’ functionality, particularly regarding effects against osteosarcoma cell lines. This type of cancer is one of the prominent malignant bone tumors in children [51]. Furthermore, bacterial infections, including those caused by MDR pathogens, are a relevant complication in the clinical outcome for patients with osteosarcoma, which defies current chemotherapy [52]. Therefore, the potent dual-action of p-Acl emerges as a cornerstone to develop therapeutics able to address both problems. 

This work gives us an additional lesson on how a single amino acid substitution may be of relevance. In particular, the role of leucine and phenylalanine residues has been the subject of other studies involving therapeutic applications of peptides [53,54,55]. In line with this, Sahoo et al. (2017) demonstrated that the substitution of leucine for phenylalanine significantly increases the toxic effect of cathelicidin-5(1-18) against cancer and bacterial cells [56]. Studies have also demonstrated the interaction of these amino acid residues with biomembranes, which is strongly dependent on the specific membrane components [54,57]. Although it is not a general principle, as evidenced by our results (for some cells, the same effect is observed for p-AppK and p-Acl, or higher activity is induced by p-AppK), this amino acid substitution may increase the capacity for targeting the membranes of certain cancer cells. Consequently, tailored Leu→Phe replacements may be considered as a useful strategy for the design and selection of therapeutic candidates, especially for osteosarcoma. However, these structure–function relationships must be understood in a holistic manner considering the diversity of the membranes and the unique organization of the peptides so that optimal peptide–membrane interactions are promoted. In this context, future investigations involving alanine scanning libraries should be useful to clarify the role of phenylalanine for the cytotoxic activity. Additionally, this antiproliferative pattern must also be evaluated at lower concentrations.

Dual targeted peptide therapy constitutes a promising treatment approach for different diseases [58]. Currently, some small molecules with antimicrobial and/or anticancer properties have been tested in clinical trials. For example, the peptide-based pharmaceuticals ANG-1005, GRN-1201, C16G2 and NP108 were evaluated by AngioChem, Green Peptide, Chengdu Sen Nuo, Wei Biotechnology and NovaBiotics, respectively [58,59]. This combination of functional roles increases the interest of the pharmaceutical market in the possible applications of the peptides [60]. For example, in oncological patients, bacterial infections are common [61]. Complications due to these infections are widespread in children diagnosed with osteosarcoma [52]. Because of this, dual peptides such as PLA_2_s-derived peptides are considered potential therapeutic candidates to this end. 

## 4. Results

### 4.1. Peptide Design and Sequence Analysis

Two venom toxin-derived peptides, p-AppK and p-Acl, were selected for this study. Both are based on Lys49-PLA_2_s isoforms from *Agkistrodon* spp. Peptide p-AppK is inspired in a membrane disrupting protein from *Agkistrodon piscivorus piscivorus*, and has been previously reported to exert cytotoxic effects on tumors. Peptide p-Acl is also derived from a membrane-damaging toxin, a Lys49-PLA_2_ from *Agkistrodon contortrix laticinctus*. The main characteristics of both peptides and their parent toxins (used as templates) are detailed in Table 1.

The bioinformatics tools employed predicted both phospholipase A_2_-derived peptides to possess antibacterial, antitumor and cell-penetrating properties. The main physicochemical parameters of both peptides, as calculated by PepDraw, are shown in Table 2. Expectedly, p-AppK and p-Acl are structurally similar and share most physicochemical characteristics, such as charge and pI. Still, they slightly differ in hydrophobicity, hydropathicity, molecular volume, and molecular weight due to leucine substitution by phenylalanine.

### 4.2. Peptide Synthesis

Both peptides were successfully produced and purified by microwave-assisted solid-phase peptide synthesis (MW-SPPS) and reverse-phase medium pressure liquid chromatography (RP-MPLC), respectively. Final purity degrees were greater than 95.5%, and the expected molecular weights were confirmed by electrospray ionization-ion trap mass spectrometry (ESI-IT MS) (Supplementary Materials, Figure S1).

### 4.3. Hemolytic Character

**Figure 1:**
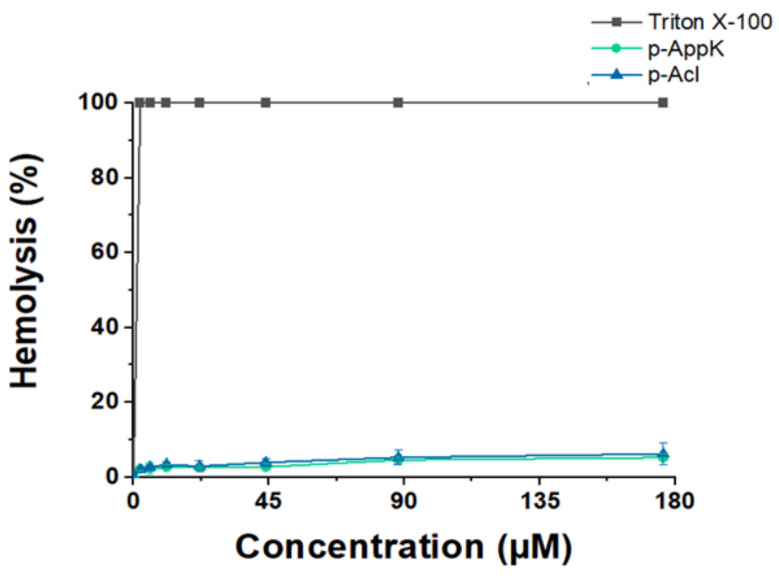
Evaluation of erythrocyte membrane disruption caused by the synthetic peptides. Red blood cells were incubated with 7 different concentrations of p-AppK (blue) and p-Acl (green). The percentage of hemolysis was measured in relation to the effect caused by a hemolytic surfactant, Triton X-100.

Functional analysis using different algorithms predicted that p-AppK and p-Acl are non-toxic peptides, with a very low probability of exerting harmful effects on red blood cells (RBCs). Data obtained in silico were corroborated by an in vitro quantitative hemolytic assay at a peptide concentration range of 2.25–176 µM (Figure 1). Triton X-100 was used as the positive control for 100% hemolysis. Minor RBC lysis was induced by the peptides, namely, below 7.0% at the highest peptide concentration tested, for both synthetic molecules. Thus, the leucine/phenylalanine substitution does not affect toxicity, suggesting a favorable safety profile for both peptides.

### 4.4. Antibacterial Activity

**Figure 2:**
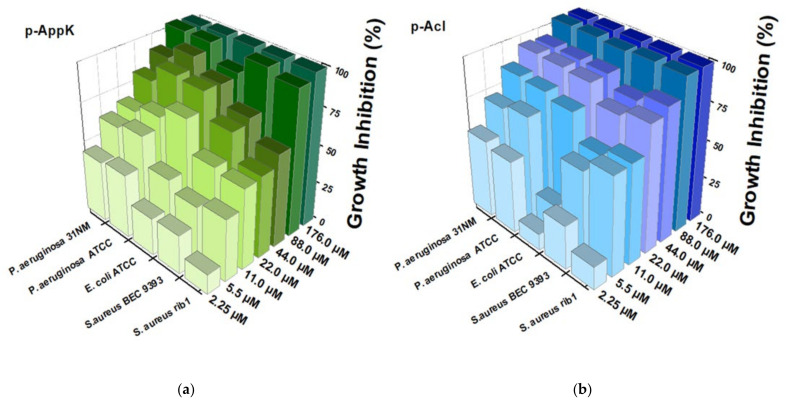
Dose-dependent inhibition of bacterial growth caused by the synthetic peptides using broth microdilution assay. (Left—(**a**)) p-AppK (green) and (Right—(**b**)) p-Acl (blue) reduced the bacterial viability (*P. aeruginosa* 31NM, *P. aeruginosa* ATCC, *E. coli* ATCC, *S. aureus* BEC 9393, and *S. aureus* rib1) after 24 h as a function of their concentrations. The growth inhibition was calculated considering the maximum optical density of the negative control as the reference. The experiments were performed in triplicate.

Peptides p-AppK and p-Acl were capable of inhibiting in vitro the growth of both Gram-positive and Gram-negative bacteria, including clinical isolates (Figure 2). *P. aeruginosa* strains (31NM and ATCC) were the most susceptible to both peptides, which showed similar antibacterial potency. The two peptides also significantly inhibited growth of *S. aureus*, but in this case p-Acl revealed a more significant effect. Interestingly, when the primary structure of p-Appk was analyzed by the Rational Design of Antimicrobial Peptides module of the CAMPR3 webserver, the sequence of p-Acl was suggested as a “potential analogous sequence”.

### 4.5. Membrane Damage

**Figure 3:**
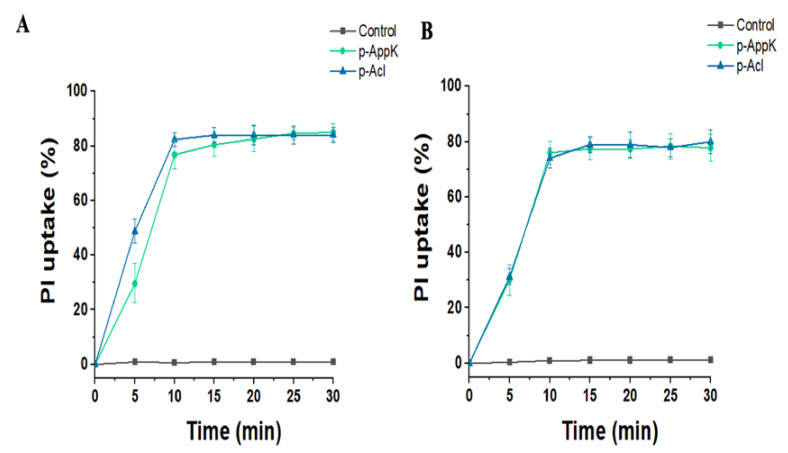
In vitro membrane-disruptive activity provoked by p-AppK (green) and p-Acl (blue). The membrane integrity evaluation of (**A**) *P. aeruginosa* ATCC and (**B**) *S. aureus* BEC9393 incubated with 100 µM peptides was determined as a function of fluorescent dye uptake.

The peptides’ effects on the membranes of two bacterial species were analyzed over time in terms of percentage of PI uptake. PI uptake remained negligible in both bacterial cultures grown in the absence of the test peptides. In contrast, when *S. aureus* and *P. aeruginosa* were grown in the presence of the peptides (100 µM), a rapid and significant increase in PI uptake was observed (Figure 3). Based on the inability of this fluorescent dye to cross intact membranes, these findings demonstrate that the peptides caused membrane damage of both Gram-positive and Gram-negative bacteria, enabling the interaction of PI with DNA.

### 4.6. Cytotoxicity

**Figure 4:**
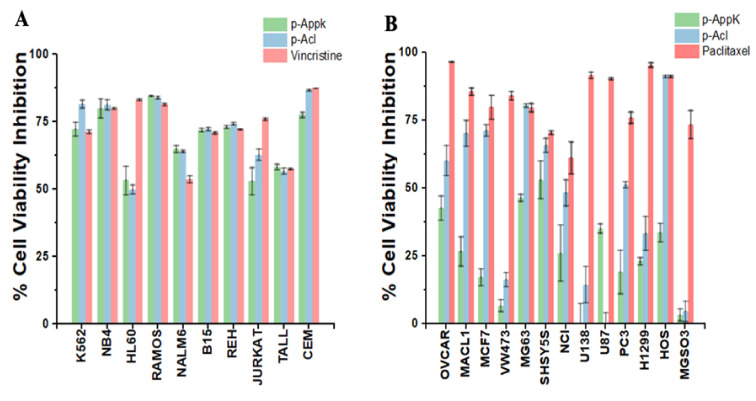
In vitro cytotoxicity of p-AppK (green) and p-Acl (blue). (**A**) Leukemia cell lines and (**B**) solid tumors were exposed to a concentration of 100 µM of both peptides. A colorimetric assay determined the cell viability inhibition after 24 h of peptide treatment. Cells cultured in a growth medium without the peptides were considered a positive control with 100% viability. The positive control (reference antineoplastic drug) is represented in salmon. The data represent the mean ± SD.

The microculture screening assay agreed with the cytotoxicity predicted by the sequence analysis software. Furthermore, at a concentration of 100 µM, the peptides showed a generalized and equipotent antiproliferative effect against most of the 10 leukemic cell lines tested (Figure 4A). Thus, the biomimetic p-AppK and p-Acl peptides were confirmed as quite promising candidates for anti-leukemic therapy, being highly toxic (≥75% cell viability inhibition) to K562, NB4, RAMOS and CEM cells.

Interestingly, though generally less potent than the reference drug against solid tumors, both peptides showed higher selectivity between the cancer cell lines tested (Figure 4B). p-Acl was consistently more efficient than p-AppK in inhibiting the viability of most cell lines. Among the cell lines susceptible to p-Acl, the reduction in the mitochondrial metabolism of human osteosarcoma lines (HOS and MG63) stands out, reaching a viability inhibition greater than 75% and like paclitaxel. These results suggest a functional impact of the p-Acl single amino acid substitution in relation to p-AppK to recognize and treat osteosarcoma. On the other hand, the effect of p-AppK is significantly lower, displaying low toxicity on these solid tumor cell lines (≤50% inhibition of viability).

### 4.7. Molecular Dynamics Simulations

Six different model membranes representative of healthy, leukemia, and thymus human cancer cells were built. In each model, peptides p-AppK and p-Acl were separately inserted, generating twelve distinct membrane–peptide systems. Interestingly, the destabilizing effect of the two peptides in the six membranes was qualitatively similar, pointing to a common permeabilization mechanism. The p-Acl-DOPS system (membrane 1) is herein described in more detail, as this was the system where the destabilizing effects were most notorious. The similar but less emphatic results for the other eleven systems are given in Supplementary Materials Figures S2 and S3. From a phenomenological point of view, both peptides induced the same kind of membrane-disturbing effect. However, the effect of peptide p-Acl was shown to be quantitatively more pronounced.

**Figure 5:**
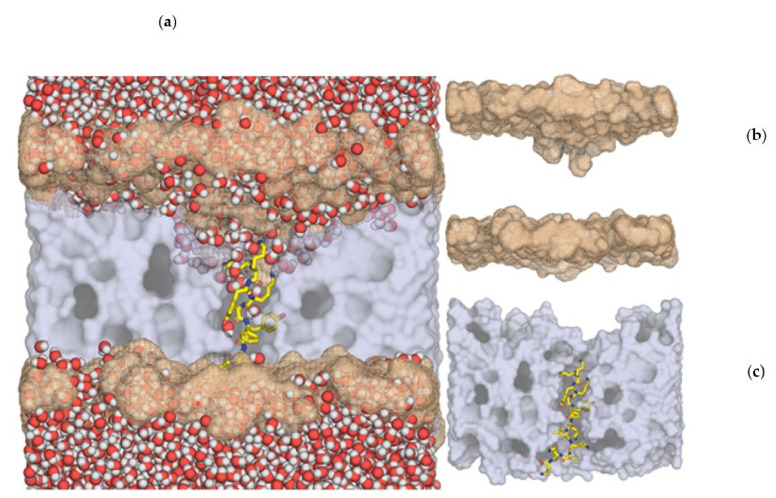
p-Acl peptide interaction with the DOPS membrane bilayer (one-half of the membrane and water molecules were removed for better visualization; the inner hydrophobic core of the membrane is shown in grey; the positions of the phosphorus atoms in the headgroups are shown in salmon). Left (**a**): a large deformation in the position of the phospholipid headgroups is visible, with the phosphate moieties penetrating deeply into the membrane core, more pronounced in the upper leaflet; in addition, the penetration of water molecules deep into the membrane is visible, confirming that the peptide induces a membrane-permeabilization effect. Top-right (**b**): only membrane inner hydrophobic core and phosphorus positions are shown, for clarity; the deformation of the membrane headgroups towards the inner part of the membrane is evident. Lower-right (**c**): insertion of peptide p-Acl (stick model) into the membrane inner hydrophobic core, showing the peptide’s perfect structural fitness to span the whole width of the membrane.

The peptides fit very well within the membrane, parallel to the phospholipids, spanning the whole membrane width. The first and last two lysines (in blue) of both peptides (KKYKAYFKLKCKK and KKYKAYFKFKCKK) established salt bridges with the phospholipid phosphate groups. The side chains of the three more central lysines (in green), place the hydrophobic region grossly parallel to the lipidic tails of the phospholipids and the ammonium group near the phosphate headgroups (Figure 5). 

In all membranes, the peptides induced a clear deformation in the bilayer, with a negative curvature emerging at both leaflets, but more pronounced in the upper one, resulting in a significant membrane thinning. For instance, the DOPS membrane width decreased by 30 Å in the center of the depression (Figure 5). This effect was the most pronounced deformation within all systems studied, as in all other cases, membrane thinning was between 15–27 Å.

**Figure 6:**
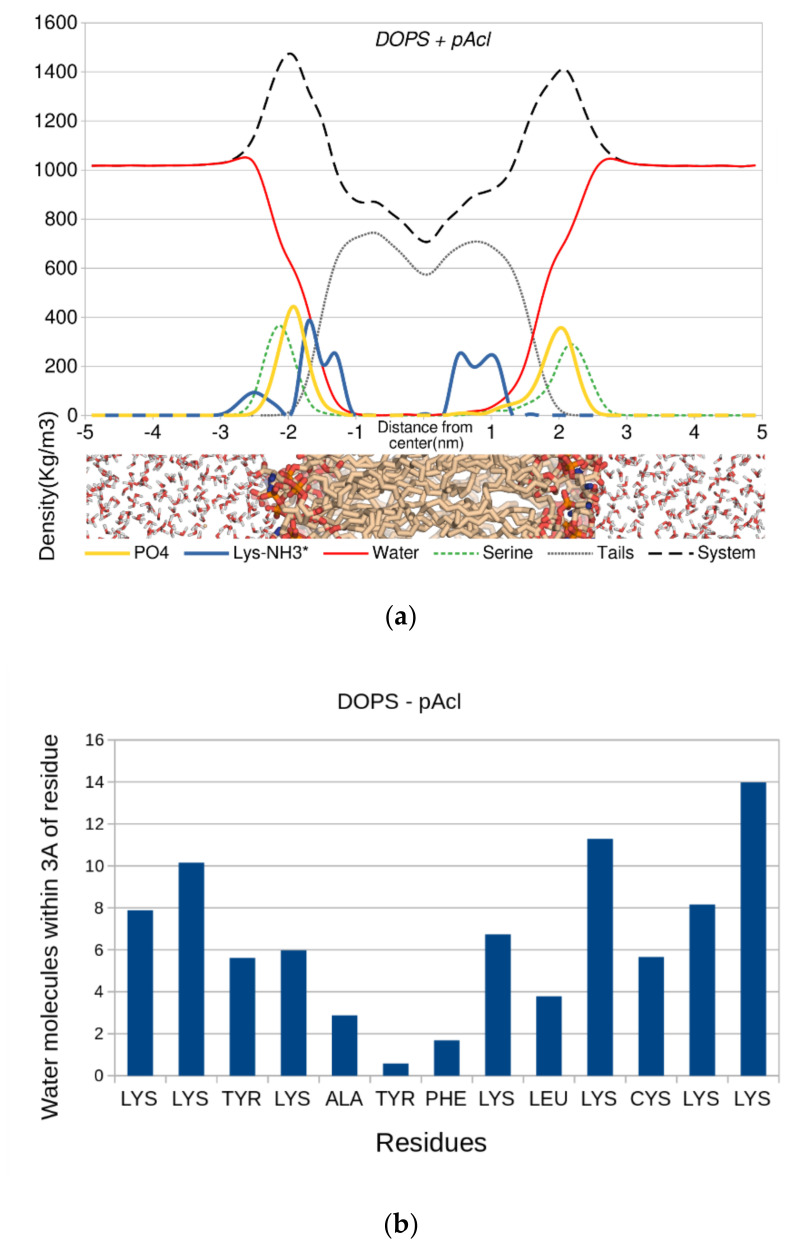
Density of the main constituents of the system as a function of the bilayer normal. Top (**a**): the center of the bilayer is located at z = 0. Bottom (**b**): the number of water molecules whose oxygen atom is within 3.0 Å of any heteroatom of the p-Acl peptide.

The density of the system was calculated as a function of the *z*-axis perpendicular to the bilayer. The origin of the axis was defined at the bilayer center (Figure 6). The profile (Figure 6a, top) showed that the distance between the phosphorus atoms and the lysine nitrogen atoms was ~5–10 Å, placing the lysine ammonium groups near or within the headgroup region, with their hydrogen atoms at close- to medium-range from the phosphate oxygen atoms. The water density inside the bilayer was small as it was averaged out throughout the whole system, not only around peptide p-Acl. The number of water molecules along the p-Acl residues is also displayed in Figure 6b (bottom). All water molecules whose oxygen atom was within 3.0 Å from the peptide heteroatoms were accounted for. The figure shows that water penetrated deep into the bilayer, with more than two water molecules close to the peptide, on average, almost up to the leaflet separation, where a stretch of three hydrophobic residues (Ala-Tyr-Phe) almost broke the water molecule chain. There was no place in the simulated system where water molecules penetrated deeply in the membrane that was not around the peptide. Hence, the increased permeability of the bilayer around the peptide was unquestionable. In the other membrane systems, an evident water penetration across the membrane was visible as well, even though it did not reach the membrane core during the simulation time (details in Supplementary Materials).

Within the sub-millisecond timescale of the simulation, the rapid penetration of water was most likely just the beginning of a chain of highly destabilizing events, which likely underpin the experimentally observed membrane permeability. Hence, the power of both peptides to induce extensive membrane weakening and permeation is clearly demonstrated both in vitro and in silico.

## 5. Conclusions

In summary, p-AppK and p-Acl showed a broad spectrum of in vitro effects, including antineoplastic and antibacterial activities in the absence of hemolysis. In general, phenylalanine favors the biological action of peptides in osteosarcoma and *S. aureus* isolates, as compared to leucine in an analogous peptide sequence. Therefore, isoforms of enzymes found in nature provide valuable information that can guide the customization of peptide drug candidates. Thus, our results validate PLA_2_ toxins with membrane-disturbing activities as a source of promising dual-target therapeutic peptides that might be useful in the development of new treatments for cancer and bacterial infections, including those caused by MDR strains.

## Figures and Tables

**Table 1:** The biomimetic peptides were designed to replicate the C-terminal region (residues 115–129) of two membrane-damaging Lys49-PLA_2_ from *Agkistrodon* spp. venoms. The molecular weight was estimated by PepCalc (https://pepcalc.com/, accessed on 1 December 2021), and the sequence identity was calculated by the Sequence Identity and Similarity tool (SIAS, http://imed.med.ucm.es/Tools/sias.html, accessed on 1 December 2021).

Peptides	Length	Molecular Weight (Da)	Parent Protein	UniProtKB	Snake Species	% Identity
p-AppK	13	1675.14	App toxin	P04361	*A. piscivorus piscivorus*	100
p-Acl	13	1709.15	Acl toxin	P49121	*A. contortrix laticinctus*	92.37

**Table 2:** Functional predictions and physicochemical parameters of the phospholipase A_2_-derived peptides. In silico tools described in the methodology section were used to predict whether the peptides had (+) or not (-) anticancer, antibacterial, and cell-penetrating peptide (CPP) properties. PepDraw was used to estimate physicochemical parameters (charge, pI, and hydrophobicity).

Peptides	Anticancer Properties	Antibacterial Properties	CPP Properties	Charge	pI	Hydrophobicity
p-AppK	+	+	+	+8	10.76	23.60
p-Acl	+	+	+	+8	10.76	23.14

## Data Availability

Not applicable.

## Supplementary Materials

The following are available online at https://www.mdpi.com/article/10.3390/cimb44010004/s1, Figure S1: Chromatographic homogeneity and mass spectrometry analysis of the synthetic peptides, Figure S2: Number of water molecules whose oxygen atom is within 3.0 Å of any heteroatom of the p-Acl and p-AppK peptides in membrane models 1-3, Figure S3: Number of water molecules whose oxygen atom is within 3.0 Å of any heavy atom of the p-Acl and p-AppK peptides in membranes 4-6, Figure S4: Representation of the DOPS model (membrane 1) used in this work with the p-Acl peptide located in the center of the bilayer (half of the membrane and water molecules were removed to visualize the peptide better), Table S1: Composition of Membrane 5 (thymocytes-like membrane model), Table S2: Composition of Membrane 6 (leukemia-like membrane model) and Table S3: Molecular dynamics minimizations and equilibrations.
