# Supplementary material for: Lessons from a Single Amino Acid Substitution: Anticancer and Antibacterial Properties of Two Phospholipase A2-Derived Peptides
Source: Curr Issues Mol Biol. 2021 Dec 22;44(1):46–62. doi: 10.3390/cimb44010004 (PMC8929095; doi:10.3390/cimb44010004)
Supplement: Supplementary file 1 [file cimb-44-00004-s001.zip › cimb-1519168-Supplementary Materials.pdf]

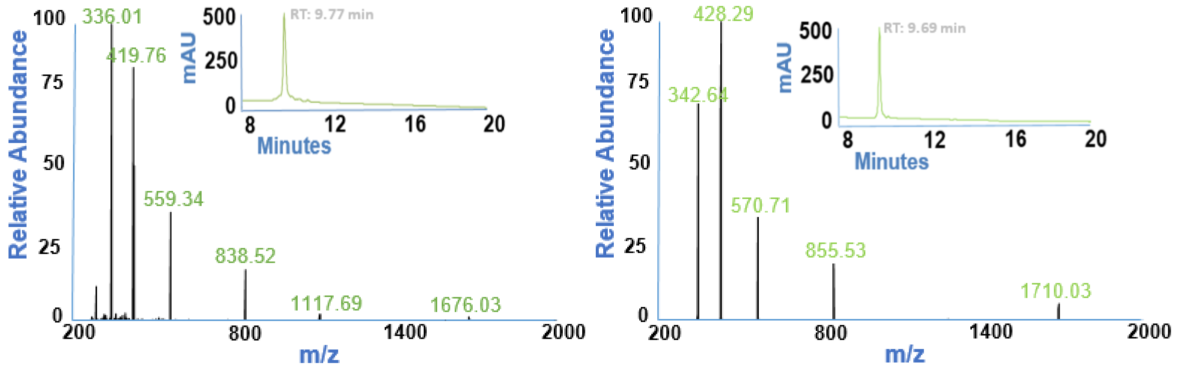


(a) (b)

**Figure S1.** Chromatographic homogeneity and mass spectrometry analysis of the synthetic peptides. Mass spectra and chromatograms of **(a)** p-AppK and **(b)** p-Acl. The peptides show high purity degrees, and their mass was confirmed by ESI-IT MS analysis.

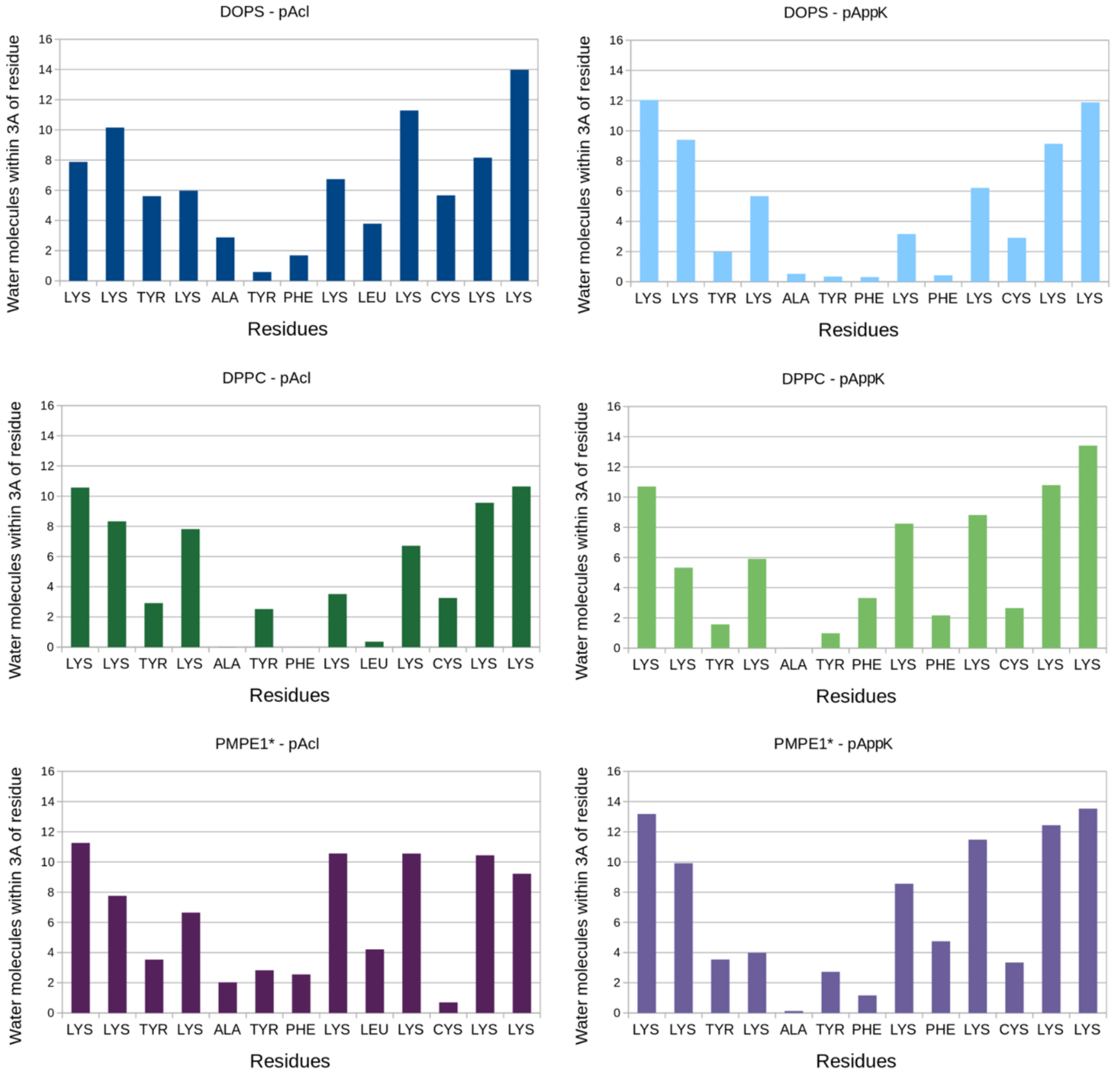


**Figure S2.** Number of water molecules whose oxygen atom is within 3.0 Å of any heteroatom of the p-Acl and p-AppK peptides in membrane models 1-3. PMPE (1*) are the most dominant phospholipids in membrane 3.

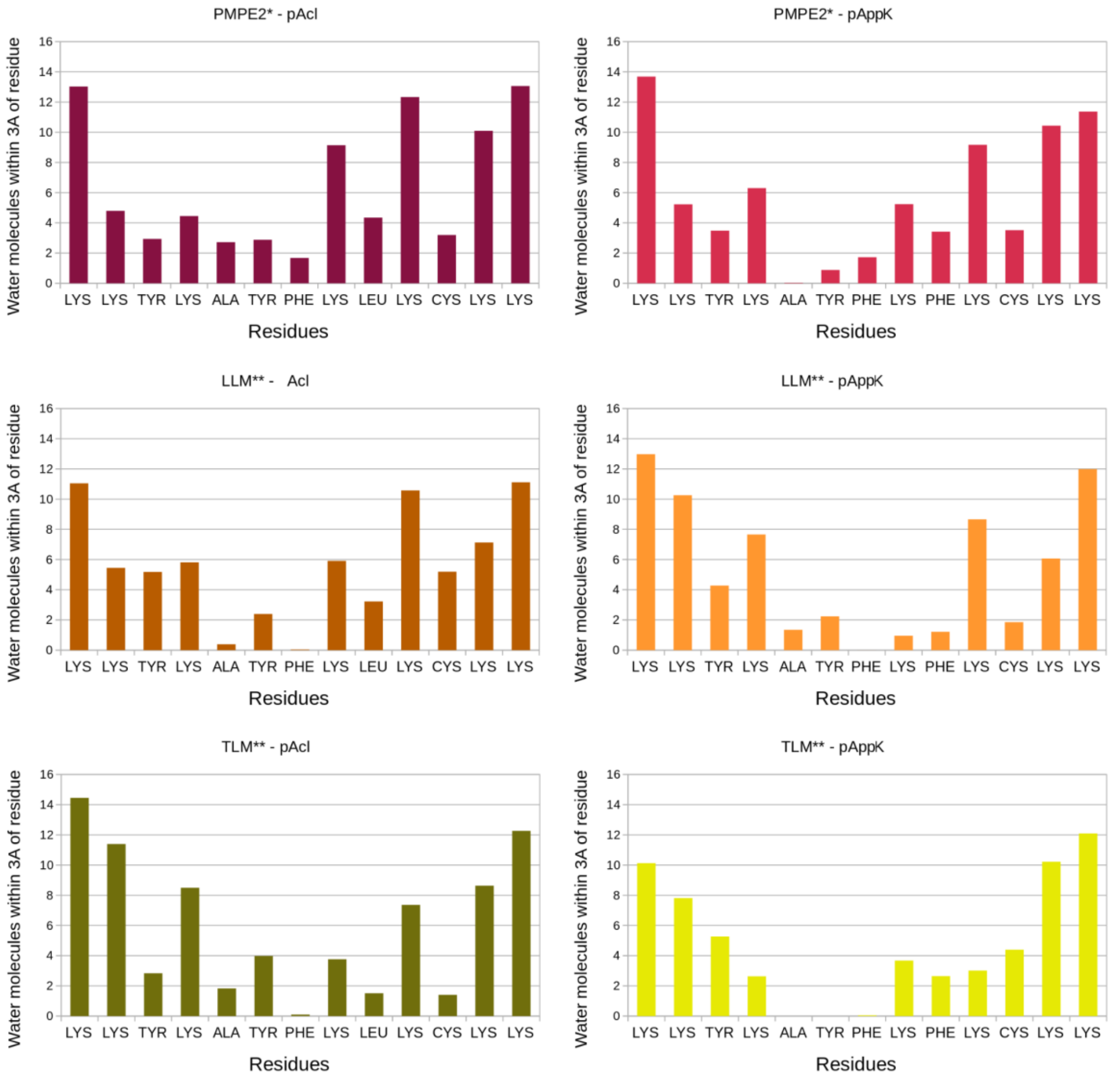


**Figure S3.** Number of water molecules whose oxygen atom is within 3.0 Å of any heavy atom of the p-Acl and p-AppK peptides in membranes 4-6. The PMPE (2*) is the most dominant phospholipid in membrane model 4. Membrane models 5 and 6 are constituted by a mixture of phospholipids, whose composition is given in tables S1 and S2; LLM** Leukemia-like membrane, TLM** Thymocytes-like membrane.

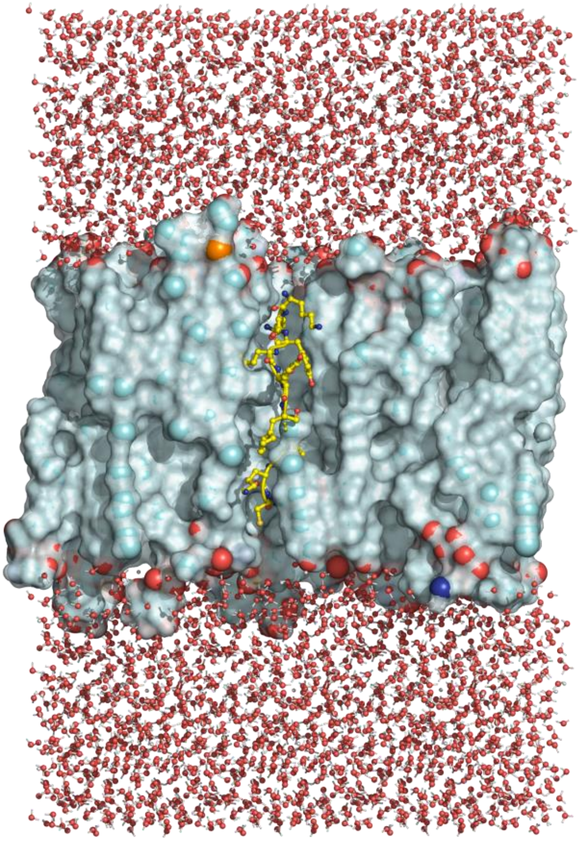

**Figure S4.** Representation of the DOPS model (membrane 1) used in this work with the p-Acl peptide located in the center of the bilayer (half of the membrane and water molecules were removed to visualize the peptide better).

**Table S1.** Composition of Membrane 5 **(**thymocytes-like membrane model)**.** The percentage of each component is given in the first column, the name of the component in the second column and the abbreviation in the third column.

| % | Name of component | Abbreviation |
|---|---|---|
| 43% | Cholesterol | |
| 20% | 2,3-dipalmitoyl-d-glycero-1-phosphatidylcholine | DPPC |
| 6% | 2,3-distearoyl-d-glycero-1-phosphatidylcholine | DSPC |
| 6% | 2,3-dioleoyl-d-glycero-1-phosphatidylcholine | DOPC |
| 4% | 2,3-distearoyl-d-glycero-1-phosphatidylethanolamine | DSPE |
| 4% | 1,2-diarachidonyl-phosphatidylethanolamine | DAPE |
| e 4% | 2,3-distearoyl-d-glycero-1-phosphatidylserine | DSPS |
| 3% | 2,3-dioleoyl-d-glycero-1-phosphatidylethanolamine | DOPE |
| 3% | sphingomyelin d18;1/16;0 | PSM |
| 3% | Phosphatidylinositol | SAPI |
| 2% | sphingomyelin d18;1/18;0 | SSM |
| 1% | 2,3-dioleoyl-d-glycero-1-phosphatidylserine | DOPS |
| 1% | 2,3 dipalmitoyl-d-glycero-1-phosphatidylserine | DPPS |

**Table S2.** Composition of Membrane 6 (leukemia-like membrane model)**.** The percentage of each component is given the first column, the name of the component in the second column and the abbreviation in the third column.

| % | Name of component | Abbreviation |
|---|---|---|
| 24% | Cholesterol | |
| 15% | 2,3-dipalmitoyl-d-glycero-1-phosphatidylcholine | DPPC |
| 10% | 2,3-distearoyl-d-glycero-1-phosphatidylcholine | DSPC |
| 9% | 2,3-dilinoleoyl-d-glycero-1-phosphatidylcholine | DUPC |
| 7% | 2,3-dioleoyl-d-glycero-1-phosphatidylcholine | DOPC |
| 7% | 2,3-distearoyl-d-glycero-1-phosphatidylethanolamine | DSPE |
| 6% | 1,2-diarachidonyl-phosphatidylethanolamine | DAPE |
| 4% | 1,2-dipalmitoyl-sn-glycero-3-phosphorylethanolamine | DLiPE |
| 4% | 1-palmitoyl-2-oleoyl-inositol | POPI |
| 3% | 1-stearoyl-2-docosahexaenoyl-phosphatidylethanolamine | SDPE |
| 3% | Phosphatidylinositol | SAPI |
| 3% | 2,3-dioleoyl-d-glycero-1-phosphatidylethanolamine | DOPE |
| 3% | Phosphatidylinositol | SAPI |
| 3% | 2,3-distearoyl-d-glycero-1-phosphatidylserine | DSPS |
| 1% | 2,3-dioleoyl-d-glycero-1-phosphatidylserine | DOPS |
| 1% | 1-linoleoyl-2-Linolenoyl-Sn-Glycero-3-Phosphatidylserine | LLPS |

**Table S3.** Molecular dynamics minimizations and equilibrations.

| **Minimizations** |
|---|
| define = -DREST_ON -DSTEP<br>integrator = steep<br>emtol = 1000.0<br>nsteps = 5000<br>nstlist = 10<br>cutoff-scheme = Verlet<br>rlist = 1.2<br>vdwtype = Cut-off<br>vdw-modifier = Force-switch<br>rvdw_switch = 1.0<br>rvdw = 1.2<br>coulombtype = pme<br>rcoulomb = 1.2<br>;<br>constraints = h-bonds<br>constraint_algorithm = LINCS |
| **Equilibrations** |
| define = -DREST_ON -DSTEP<br>integrator = md<br>dt = 0.002<br>nsteps = 50000<br>nstlog = 1000<br>nstxout = 1000<br>nstvout = 1000<br>nstfout = 1000<br>nstcalcenergy = 100<br>nstenergy = 1000<br>;<br>cutoff-scheme = Verlet<br>nstlist = 20<br>rlist = 1.2<br>coulombtype = pme<br>rcoulomb = 1.2<br>vdwtype = Cut-off<br>vdw-modifier = Force-switch<br>rvdw_switch = 1.0<br>rvdw = 1.2<br>;<br>tcoupl = berendsen<br>tc_grps = PROT MEMB SOL_ION<br>tau_t = 1.0 1.0 1.0<br>ref_t = 303.15 303.15 303.15<br>; |

```
pcoupl = berendsen
pcoupltype = semiisotropic
tau_p = 5.0
compressibility = 4.5e-5 4.5e-5
ref_p = 1.0 1.0
;
constraints = h-bonds
constraint_algorithm = LINCS
continuation = yes
;
nstcomm = 100
comm_mode = linear
comm_grps = PROT MEMB SOL_ION
;
refcoord_scaling = com
```
